# Characterization of Different Microbubbles in Assisting Focused Ultrasound-Induced Blood-Brain Barrier Opening

**DOI:** 10.1038/srep46689

**Published:** 2017-04-20

**Authors:** Sheng-Kai Wu, Po-Chun Chu, Wen-Yen Chai, Shih-Tsung Kang, Chih-Hung Tsai, Ching-Hsiang Fan, Chih-Kuang Yeh, Hao-Li Liu

**Affiliations:** 1 Institute of Biomedical Engineering, College of Medicine and College of Engineering, National Taiwan University, Taipei, Taiwan; 2 Department of Research and Development, NaviFUS corp., Taipei, Taiwan; 3 Department of Electrical Engineering, Chang-Gung University, Taoyuan, Taiwan; 4 Department of Diagnostic Radiology and Intervention, Chang-Gung Memorial Hospital, Taoyuan, Taiwan; 5 Department of Biomedical Engineering and Environmental Sciences, National Tsing Hua University, Hsinchu, Taiwan; 6 Department of Neurosurgery, Chang Gung Memorial Hospital, Taoyuan, Taiwan; 7 Medical Imaging Research Center, Institute for Radiological Research, Chang Gung University and Chang Gung Memorial Hospital, Taoyuan, Taiwan

## Abstract

Microbubbles (MBs) serve as a critical catalyst to amplify local cavitation in CNS capillary lumen to facilitate focused ultrasound (FUS) to transiently open the blood-brain barrier (BBB). However, limited understanding is available regarding the effect of different microbubbles to induce BBB opening. The aim of this study is to characterize different MBs on their effect in FUS-induced BBB opening. Three MBs, SonoVue, Definity, and USphere, were tested, with 0.4-MHz FUS exposure at 0.62–1.38 of mechanical index (MI) on rats. Evans blue, dynamic contrast-enhanced (DCE) MRI and small-animal ultrasound imaging were used as surrogates to allow molecule-penetrated quantification, BBB-opened observation, and MBs circulation/persistence. Cavitation activity was measured via the passive cavitation detection (PCD) setup to correlate with the exposure level and the histological effect. Under given and identical MB concentrations, the three MBs induced similar and equivalent BBB-opening effects and persistence. In addition, a treatment paradigm by adapting exposure time is proposed to compensate MB decay to retain the persistence of BBB-opening efficiency in multiple FUS exposures. The results potentially improve understanding of the equivalence among MBs in focused ultrasound CNS drug delivery, and provide an effective strategy for securing persistence in this treatment modality.

The blood-brain barrier (BBB) plays an important role in regulating the exchange of nutrients and wastes between brain tissues and the circulatory system while concurrently preventing pathogens from entering the brain parenchyma. The BBB structure encompasses occludins, claudins, and junctional adhesion molecules to support specialized endothelial cells, forming the tight junction to prevent molecules larger than 400 Da from entering the brain^1^. The BBB allows for the controlled transport of necessary peptides and proteins to maintain proper neuronal function, even under pathological conditions. It poses a critical impediment to the pharmaceutical treatment of certain brain diseases.

Low-pressure burst mode focused ultrasound (FUS) exposure in conjunction with the administration of microbubbles (MBs) has been found to produce transient alteration of BBB permeability^2^. This technology has the potential to enhance delivery of various kinds of therapeutic agents into the brain and has potential to benefit treatment of CNS diseases. A growing number of applications have been developed for the use of therapeutic ultrasound in preclinical investigation, such as neuroprotectant delivery for ischemia-reperfusion brain injuries^3^, antibody delivery for treatment of Alzheimer’s disease^4^, gene delivery for Huntington’s and Parkinson’s diseases^5^,^6^, and chemotherapeutics for brain tumors^7^,^8^,^9^,^10^. Circulating microbubbles passing through ultrasound exposure encounter acoustic-actuated harmonic extraction/expansion, with the produced microstreaming and radiation force providing physical stimulation to the endothelial cells and then cause temporal tight junction disruption^11^,^12^.

Microbubbles play a critical role in catalyzing the FUS-induced BBB-opening effect. At present, commercialized MBs include Optison^TM^ (GE Healthcare, WI, USA), Definity^®^ (Lantheus Medical Imaging, MA, USA), and SonoVue^®^ (Bracco, Milano, Italy), all with clinically approved for diagnostic purposes by the FDA. Commercial bubbles are generally larger than 2 μm and their applicable time window is about 5–10 min. These commercialized MBs have been shown to effectively assist with the opening of the BBB under FUS exposure^5^,^13^,^14^,^15^. With a smaller mean size (about 1 μm), USphere (Turst Inc., Taiwan) is a new commercialized MBs currently in clinical trials, but its isoform has already been shown to effectively assist in BBB opening^16^,^17^. The composition, concentration, half-life and hydrodynamic sizes of these MBs are all distinct, raising a critical concern regarding how MBs differences affect MBs-ultrasound interaction on CNS capillary permeability changes. However, the effects of various commercialized MBs on FUS-induced BBB opening are not sufficiently understood.

In this study, we compared three different microbubbles – SonoVue, Definity, and USphere in terms of their effect on BBB opening when combined with FUS. To characterize the effect of various MBs on FUS-induced BBB opening, we conducted FUS exposure *in-vivo* animal studies with different MBs and tested the BBB-opening effects under different ultrasound exposure levels while maintaining injected bubble concentrations. To test BBB-opening endurance of different MBs, multiple FUS exposures were used to determine the effective persistence of BBB-opening under a bolus MBs injection. We also attempted to determine whether FUS exposure time adaptation can compensate for MB circulation decay to improve BBB-opening persistence. Imaging including small-animal diagnostic ultrasound and MRI was used to confirm MB dynamics and BBB-opening effect *in-vivo*. Potential erythrocyte extravasations were also examined histologically. Cavitation activity was measured via passive cavitation detection (PCD) to correlate with the exposure levels and the histological effects.

## Results

### *In-vitro* characterization of three MBs via passive cavitation detection

**Figure 1:**
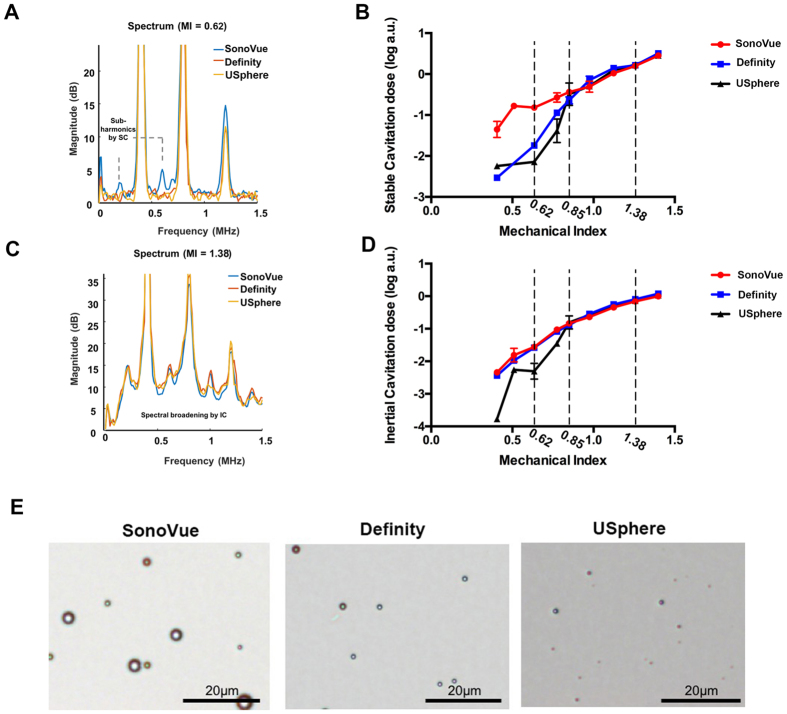
(**A**) Typical frequency spectrum acquired from PCD during 0.4-MHz sonication with different microbubbles at 0.62 MI. (**B**) Stable cavitation dose measurement acquired following different exposure levels using different microbubbles. (**C**) Typical frequency spectrum acquired from PCD during 0.4-MHz sonication with different microbubbles at 1.38 MI. (**D**) Inertial cavitation dose measurement acquired at different exposure levels using different microbubbles. (**E**) Microscopy images of three different microbubbles (SonoVue, Definity, and USphere). Scale bar = 20 μm.

*In-vitro* PCD analysis was conducted to compare the cavitation activity induced by different MBs at various FUS exposure levels. Figure 1 shows the spectra of acoustic-emission detections and the exposure-dependent SCD/ICD. Figure 1(A) and (C) demonstrates the emission spectra comparing the three MBs for FUS exposure levels of 0.62 and 1.38 MI (n = 5 per exposure condition). The SonoVue-group generated more apparent subharmonic/ultraharmonic emissions at low (0.62 MI) exposure level (2–3 dB higher than the Definity- and USphere-group), indicating that stable cavitation activity in SonoVue can be easily triggered at this exposure level (these subharmonic/ultraharmonic emissions were all triggered in three MBs at 1.38-MI exposure). On the other hand, wideband emissions were not apparent in 0.62-MI exposure for three MBs, but were all significantly elevated at 0.85-MI (or above) exposure level, implying the inertial cavitation did not present at 0.62-MI but were significantly involved at high (1.38-MI) exposure. When transferring the data to SCD/ICD indexes (see Fig. 1(B) and (D)11-Apr-2017 12:2811-Apr-2017 12:2811-Apr-2017 12:2811-Apr-2017 12:28; n = 5 per exposure condition), spectrum observation showed that the cavitation behavior of the three MBs tended to be identical at high FUS exposure level (higher than 0.85 MI). However, SonoVue tended to present stronger stable cavitation whereas USphere tended to suppress the inertial cavitation at low (0.62 MI) FUS exposure levels.

### *In-vivo* characterization of three MBs via small-animal ultrasound contrast imaging

**Figure 2:**
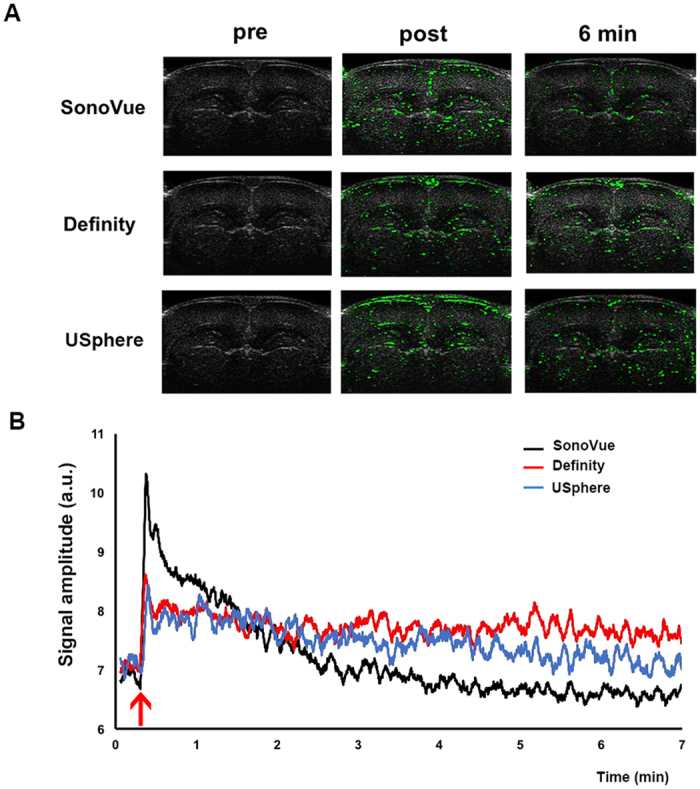
(**A**) Representative contrast signal (green color) images were taken before and after injection of identical amounts of three different microbubble types. (**B**) Signal intensity change of three microbubbles over time. The arrow indicates the MB injection time.

Figure 2(A) illustrated typical brain contrast images before and after three MBs injections to measure the circulation persistence of the three MBs in the animal brain (with contrast change caused by MBs circulation encoded and presented in green in the B-mode image; n = 3 per group). The post-injection images showed an immediate enhancement among these three MBs administrations. A comparison of the contrast-enhanced encoded levels immediately following and 6-min after MB administration showed MB-induced contrast changes reached a maximum and then rapidly decayed. The SonoVue-group presented the greatest signal enhancement, which can be explained by the larger bubble size contributing to a more significant imaging contrast change than the other two microbubble types. However, the SonoVue-group also had the greatest contrast decline. Figure 2(B) showed the contrast signal changes over time. The half-life of SonoVue calculated from the contrast-encoded change was estimated to be 1.04 ± 0.15 min (n = 3). As measured in the images, the contrast-based half-life values due to Definity and USphere administration were respectively estimated to 6.88 ± 4.88 min. and 4.98 ± 0.83 min (both n = 3), which were both slightly longer than the estimated value of SonoVue.

### Characterization of three MBs on efficiency/multiple-exposure persistence of BBB-opened induction

**Figure 3:**
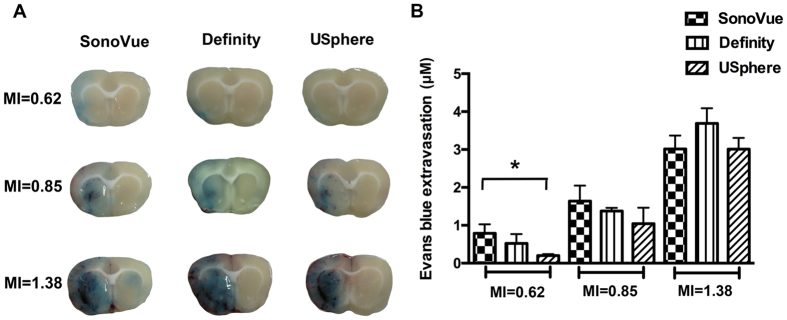
*In vivo* BBB opening using different microbubbles at various exposure levels. (**A**) Representative EB images indicated the degree of BBB opening. (**B**) EB extravasation was used to quantify the BBB opening (n = 3 per subgroup, totally 3 subgroups).

To investigate the efficiency of FUS-induced BBB-opening in conjunction with SonoVue, Definity, and USphere administration, animal brains were subjected to a 2-min single FUS exposure (MI = 0.62, 0.85, and 1.38; n = 3 per group) with quantified Evans blue (EB) leakage and staining when fixing the injected microbubble concentration to be 4 × 10^7^ bubbles/kg. Figure 3(A) showed representative EB stained brain sections, while Fig. 3(B) quantified the EB penetrated concentrations. At the 0.62-MI exposure level, all three MB types successfully induced BBB-opening. The SonoVue groups produced EB penetration rates significantly higher than USphere (0.79 ± 0.24 vs. 0.2 ± 0.04 μm; *p* = 0.04) and was slightly higher than Definity (0.79 ± 0.24 vs. 0.52 ± 0.25 μm; *p* = 0.4), whereas Definity was also found to be slightly higher than USphere (*p* = 0.28). The 0.85-MI exposure induced similarly elevated EB penetration among all three MBs groups (1.64 ± 0.41, 1.37 ± 0.08, and 1.04 ± 0.42 μm respectively in the SonoVue-, Definity-, and USphere-groups; *p* > 0.05 between each group comparison). Increasing exposure levels up to 1.38 MI not only reached the highest EB penetration in all exposure levels (3.01 ± 0.35 μm, 3.69 ± 0.4 μm, and 3.01 ± 0.29 μm respectively in the SonoVue-, Definity-, and USphere-groups; *p* > 0.05 between each group comparison), but also induced apparent and large-scale erythrocyte extravasation that can be directly observed from brain sections from the three groups (described below).

**Figure 4:**
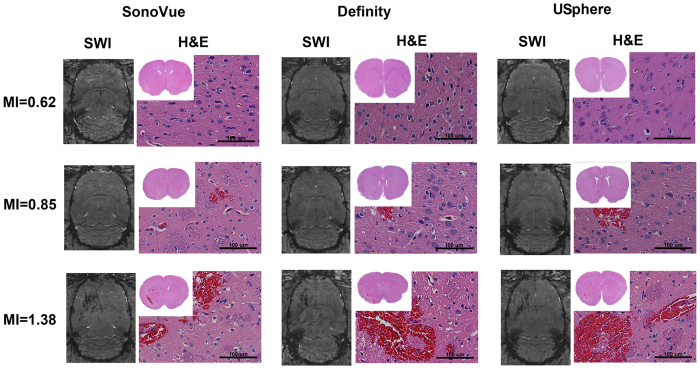
MR-SWI images and their corresponding HE-stained brain sections at different MI levels for three MB administrations. Magnification is 40X and scale bar is 100 μm.

Figure 4 showed the corresponding MR-SWI and HE stained examinations of Fig. 3 to detect histological changes, particularly for erythrocyte extravasations. Susceptibility weighted imaging (SWI) was used to evaluate the presence of apparent erythrocyte extravasation caused by FUS-induced BBB opening among the three MB types. The 0.62-MI exposure induced neither MR-SWI signal changes nor erythrocyte extravasations for any of the MB groups. Occasionally small-scale extravasated erythrocytes (a regional dimension < 100 μm) were histologically observed at the 0.85-MI exposure site, but these results still did not meet the detection range via MR-SWI for all three MB groups, implying the extravasations were minimal. Exposure at 1.38-MI not only caused a noticeable signal drop observed from MR-SWI signal changes for all MBs groups, but also resulted in large-scale erythrocyte extravasations (regional dimension > 100 μm).

**Figure 5:**
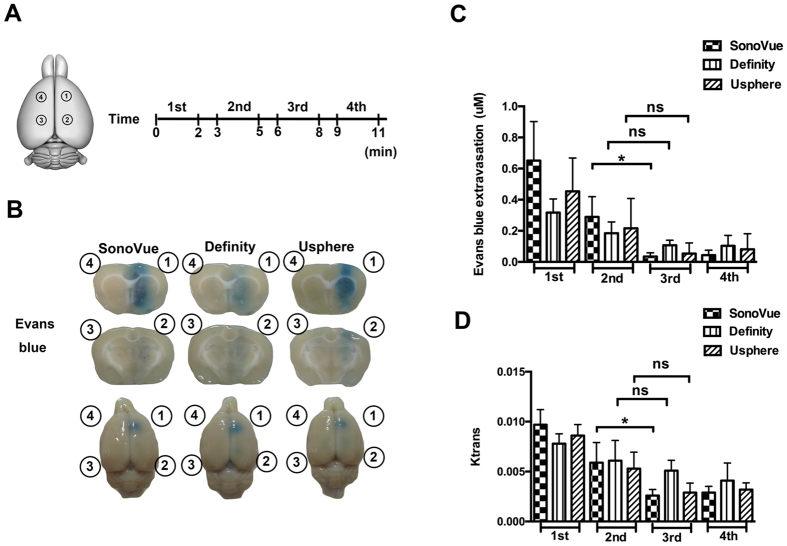
(**A**) Diagram of the sonicated location and the time course of sonication in multiple-exposure persistence test when fixing the exposure time at each target site. Four exposures of 2-min duration with 1 min interval in between were executed. (**B**) Representative EB images were shown to indicate the ability of the different microbubbles to open the BBB at multiple sites. (**C**) Time-dependent changes were shown in EB penetration at different sonicated locations (n = 5 per subgroup, for 3 subgroups). (**D**) K_trans_ value was shown to estimate the BBB opening at different sonicated locations.

We then tested the persistence of inducing multiple-exposure BBB-opening over time under a single-bolus administration for the three MBs. Four FUS exposures were sequentially applied (0.62 MI, 2 min per exposure, with a concomitant 1-min. interval prior to next exposure) in a clockwise order with a 5-mm space between individual exposures (n = 5 per group; see Fig. 5(A)). Representative top and coronal views of the brains were shown, confirming BBB opening by EB extravasation (Fig. 5(B)). For all three MBs groups, the 1^st^ exposure induced the most profound BBB-opening effect. The 2^nd^ exposure also induced opening, but the EB-stained effect in the brain section was faded, implying significant decay of the BBB-opening effect. The 3^rd^ and 4^th^ exposures did not induce noticeable EB-staining on brain, indicating further decay. Figure 5(C) showed the EB quantification measured from the dissected tissues at four exposure sites. The SonoVue group presented the most significant decay of the BBB-opened effect. The EB concentration in the SonoVue-group had a 57% concentration decay (from 0.65 ± 0.25 to 0.28 ± 0.13 μm) in the 2^nd^ exposure, and dropped to 95% of the EB concentration in the 3^th^ exposure (0.03 ± 0.02 μm). The EB concentration was relatively low in the 1^st^ exposure of the Definity- and USphere-groups (0.32 ± 0.09 and 0.45 ± 0.21 μm, respectively), but with relatively lower measured concentration decay in the 3^rd^ exposure (68% and 88%, respectively), implying slightly superior BBB-opening persistence as compared to SonoVue (group effect of F = 1.657; *p* = 0.201). The EB penetration sharply declined along the multiple exposures (group effect of F = 26.538; *p* < 0.001). Figure 5(D) showed the estimated K_trans_ from the exposure location, with a decline trend similar to that observed in Fig. 5(C). When fixing the exposure time to be 2-min., all microbubble groups did not persist the BBB-opened effect for all exposures (group effect of F = 0.701; *p* = 0.506), whereas the K_trans_ differed significantly across the multiple exposures (group effect of F = 20.795; *p* < 0.001).

### Adaption of FUS exposure to improve multiple-exposure BBB-opened persistence

**Figure 6:**
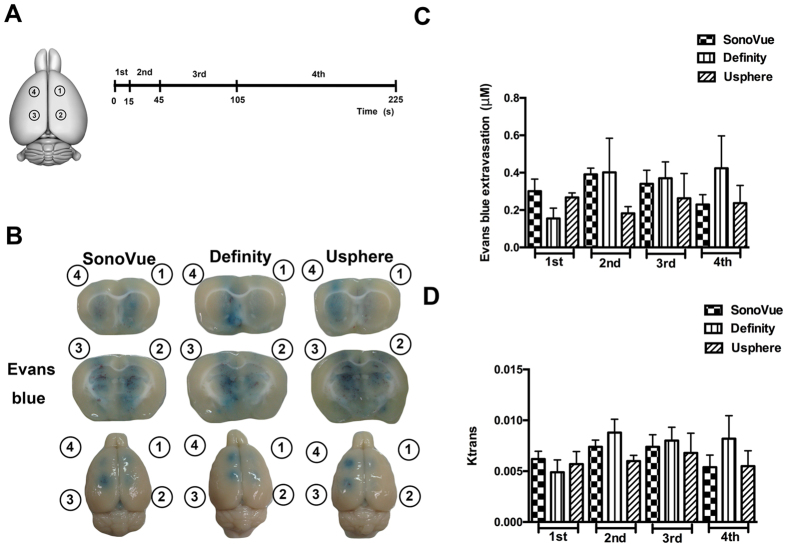
(**A**) Diagram of the sonicated location and the time course of sonication in multiple-exposure persistence test when adjusting the exposure time at the 4 target sites. Exposure times for the 4 targets were set to 15, 30, 60, and 120 s, respectively. (**B**) Representative EB images were shown to indicate the ability of different microbubbles to open the BBB at multiple sites. (**C**) Time-dependent changes of EB penetration were displayed at different sonicated locations (n = 5 per subgroup, for 3 subgroups). (**D**) K_trans_ value was shown to *in-vivo* estimate the BBB opening at different sonicated locations.

The above showed constant exposure-time scheme was unable to maintain a persistent BBB-opening effect. Therefore, an adapted design using four increasing exposure times (i.e. 15, 30, 60, and 120 s; no time lapse between exposure) at a fixed 0.62-MI exposure level was proposed to verify the feasibility of compensating for geometrically-decreased MB concentrations in circulation (n = 5 per each group; see Fig. 6(A)). Figure 6(B) showed the representative EB-stained brains from experiments conducted using the three MB types. Under the same single bolus MB injection, apparent EB penetrations were observed even at the 4^th^ exposure location for all three MB groups, indicating this geometrically increased exposure time successfully compensated for the degradation of MB concentrations in circulation over time.

Figure 6(C) shows the EB quantification measured from the dissected tissues obtained from the four exposure sites. When adjusting the exposure time, all microbubble groups persisted the BBB-opened effect for all exposures (group effect of F = 1.237; *p = *0.302), whereas the EB distributions didn’t show sharp changes across the exposures (group effect of F = 0.518; *p = *0.673). The SonoVue group had a relatively even EB distribution, whereas the EB concentration at the 1^st^ exposure site was measured to be 0.3 ± 0.06 μm, whereas the 4^th^ exposure site still maintained to be 0.23 ± 0.05 μm without decay. In the Definity group, an enhanced BBB-opening persistence was also observed, with the EB concentration at the 1^st^ exposure site measured to be 0.15 ± 0.06 μM and a slight increase measured to be 0.42 ± 0.17 μm at the 4^th^ exposure site. In the USphere group, the EB concentration at the 1^st^ exposure site was measured to be 0.27 ± 0.02 μM, whereas the 4^th^ exposure site was measured to have a similar level of 0.24 ± 0.1 μm.

Figure 6(D) showed the K_trans_ levels in the four exposure sites. The K_trans_ value corresponding to the four sonicated regions were also analyzed to show the BBB opening over time. When adjusting the exposure time, all microbubble groups presented different BBB-opened effect for all exposures (group effect of F = 3.737; *p < *0.05), whereas the K_trans_ differed across the multiple exposures (group effect of F = 0.3807; *p < *0.05). In the SonoVue group, the K_trans_ at the 1^st^ exposure site was 0.0062 ± 0.0008 min^−1^, and can be maintained to 0.0054 ± 0.0012 min^−1^ at the 4^th^ exposure site. In Definity group, not only an improved BBB-opening persistence but also gradual permeability increase (K_trans_ estimated from 0.005 ± 0.001 min^−1^ to 0.008 ± 0.002 min^−1^ from the 1^st^ to 4^th^ exposure), implying a relatively long persistence of Definity than the other two microbubbles. In the USphere group, the degree of BBB-opening persistence was similar to the SonoVue group (with K_trans_ changing from 0.0057 ± 0.0012 min^−1^ to 0.0055 ± 0.0015 min^−1^ from the 1^st^ to 4^th^ exposures without decay). Figure S2 showed the corresponding MR-SWI as well as HE staining results, indicating that this exposure-time adjusting scheme can uniformly open the BBB for all 4 exposures but did not induce noticeable erythrocyte extravasations.

**Figure 7:**
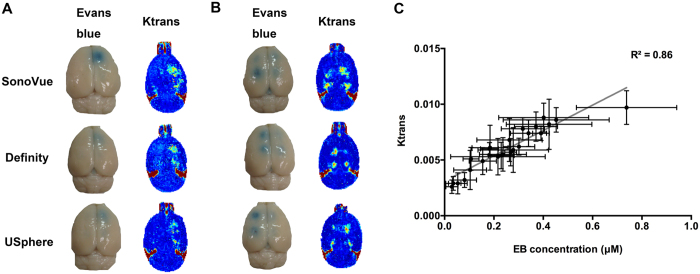
Typical Evans blue stained brains and representative Ktrans maps at the same exposure condition were utilized to show multiple exposures when (**A**) fixing exposure time to 120 s and (**B**) adjusting exposure-time to 15, 30, 60, and 120 s during various microbubble usage. (**C**) Correlation between K_trans_ and measured EB concentrations.

To further understand the relationship between EB extravasation and K_trans_, we compared the measured EB concentration with the K_trans_ level (data was presented as mean ± standard deviation for each subgroup; n = 5 per subgroup, for totally 24 subgroups (12/12 in exposure-time fixed/adjusted group, respectively)). Figure 7(A) and (B) showed typical images of EB stained brains and K_trans_ maps reconstructed from DCE-MRI in the multiple exposures persistence tests when exposure-time was being fixed and adjusted, respectively. The overall correlation between EB concentration and K_trans_ was observed (Fig. 7(C); r^2^ = 0.86), indicating that DCE-MRI provides a qualified tool for measuring dynamic change in the *in-vivo* BBB-opening, and also implied potential to serve as an alternative for *ex-vivo* pharmacodynamic analysis for FUS induced BBB-opening.

## Discussion

This study compares and characterizes the efficiency of FUS-induced BBB opening using three different microbubbles. The three tested MBs (SonoVue, Definity, and USphere) were all found to be able to induce the BBB-opened effects, and the quantitated surrogate molecule penetration were observed to be comparable, implying that the BBB-opening effect might be predictable, generalized, and MB-independent given carefully controlled MB dose administration. The persistence of the BBB-opening effect was found to be dependent on the degradation dynamics of each MB type. We also found that a simple exposure adaptation can compensate for MB degradation to produce a more persistent BBB-opening effect by delivering multiple exposures to cover a large treatment volume. These results might contribute to further optimization of the FUS-induced BBB opening procedure using various MB types, and may help to improve the quality of FUS-induced CNS drug delivery.

Although three microbubbles produced comparable BBB-opened effect, SonoVue provided relatively superior BBB opening performance than Definity and USphere at low MI (i.e., 0.62) exposure. FUS-induced BBB opening in the presence of MBs can be explained by several physical mechanisms, but stable cavitation (as measured by SCD) is one of the key factors contributing to this phenomenon. Figure 1 showed a higher SCD while using SonoVue at low (0.62 MI) sonication, which was correlated to the finding in EB quantification shown in Fig. 3B. In addition, no SCD differences were found at 0.85- and 1.38-MI exposure levels and apparent ICD elevations were accompanied for all MBs, implying that inertial cavitation (IC) effect was strongly involved. IC has been found to possibly cause erythrocyte extravasations or brain damage at high pressure sonication^13^,^15^. As the observation shown in Fig. 1(C), all three MB types displayed similar wideband acoustic emissions at 1.38-MI exposure, which might lead to some damage (see Fig. 4). Some slight damage was found at 0.85-MI exposure and mass erythrocyte extravasations formed when the MI increased to 1.38, implying the exposure level should be carefully controlled to assure exposure safety.

This study investigates the effect of administering different types of microbubbles on the persistence of FUS-induced BBB opening. SonoVue was estimated to have a 1.04 min. circulation half-life based on ultrasound imaging contrast change. This estimation was found to be consistent with previous results (~1 min.)^18^. SonoVue had a higher signal peak immediately after bolus administration but a faster rate of decay than Definity and USphere. The stronger enhancement was due to the larger microbubbles more easily interacting with ultrasound energy^19^. This might explain that, under identical MB concentrations and at moderate exposure levels (e.g., 0.62 MI), SonoVue contributed to higher SCD activity (see Fig. 1(B)) as well higher EB penetration (see Figs 3(B) and 5(C)).

On the other hand, Definity and USphere were found to have longer circulation half-lives (4.98–6.88 min.). This prolonged elimination time for Definity and USphere can explain the observation in Fig. 5(C). The SonoVue group showed the lowest EB penetration at the 3^rd^ and 4^th^ exposure, whereas slightly enhanced EB penetration can be still observed in the Definity and USphere groups. Small-animal ultrasound imaging showed a reduced contrast enhancement for Definity and USphere, which might also be due to their relatively smaller MB size making them less efficient in reflecting high backscattered ultrasound signals for analysis, or the thresholding imaging processing algorithm built into the imaging system console may potentially contribute to signal-intensity decay.

For the exposure-time adaptation experiments, the exposure time for each point was sequentially increased in an increased fashion as a result of a geometric degradation of the circulating MBs in Fig. 2. Instead of using EB quantification, the permeability of coefficient K_trans_ can well reflect the pharmacodynamic change of the MR tracer during the BBB-opening process based on previous studies showing a high correlation between DCE-MRI based pharmacodynamic/pharmacokinetic analysis and *ex-vivo* histological analysis^20^,^21^. We observed that SonoVue can uniformly enhance the distribution of EB at the four exposure levels. However, Definity and USphere showed a slight EB-elevated trend, implying that an exposure time adaption did not optimally compensate all MB degradations. This might be due to that Definity and USphere have longer circulation lives (4.98–6.88 mins.) than SonoVue, thus the current exposure time adaption can well fit SonoVue but overcompensates for the exposure time for Definity/USphere. This also implies that the FUS exposure time adaptation should be custom-designed for individual MBs when BBB-opening uniformity is intended.

Previous reports characterizing three different MBs (Optison, Definity, and Imagent) in rat heart images found no significant differences resulting from the administration of similar numbers of microbubbles^22^. These results indicated that encapsulated gas, as well as shell type, rarely affect their bioeffects. BBB opening with nonlinear bubble oscillation has been shown to be highly dependent on both acoustic pressure and bubble diameter^23^. However, commercialized microbubbles vary in terms of size and distribution. SonoVue has a mean bubble diameter of around 2.5 μm and more than 90% of the bubbles are smaller than 8 μm, while the mean diameter of Definity microbubbles is around 1.1 μm to 3.3 μm. (Figure S3 shows microbubble size and distributions characterized using a method described in ref. 17). Attenuation measurements have been used in a number of studies at lower frequencies. SonoVue was found to have an attenuation peak at 1.5 to 2 MHz^24^ while that of Definity was 2 to 6 MHz^25^. In this study, a 0.5-MHz transducer was used to sonicate microbubbles to induce BBB opening, and larger bubbles are potentially more sensitive to this frequency. Hence, a higher stable cavitation signal was detected in the SonoVue group at low power exposure (0.62 MI) as compared with Definity and USphere. Interestingly, no obvious damages were found at this exposure level with these three microbubbles. For now, stable cavitation dominates the BBB opening at the pressure threshold and can be used as an indicator to control the quality of BBB opening^26^,^27^. Our results suggest that different microbubbles might result in various degree of signals. Further investigation is needed when different microbubbles were employed to perform a signal feedback for BBB opening control.

## Materials and Methods

### Microbubbles Characterization

Microbubbles (MBs) were constituted according to the manufacturer’s guidelines. Based on concentrations (Table 1) suggested by the manufacturer. The size distribution, number, and volume analysis of the three microbubbles were characterized with the approach that previously reported^17^. The regional distribution and the diameter of MBs was optically observed with an inverted microscope (model IX71, Olympus Co., Tokyo, Japan) at 200X magnification (typical observation of three microbubbles see Fig. 1(E); Measured MBs size distribution see Fig. S3).

### Animals

All animal protocols were approved by the Institutional Animal Care and Use Committee of Chang-Gung University and adhered to the experimental animal care guidelines. A total of 66 male Sprague-Dawley rats (250–300 g) were used: 9 rats for microbubble half-life testing, 27 rats for the MI-dependent BBB-opened effect testing, and 30 rats for multiple sonication BBB-opened persistence testing. Detailed animal number conducted in this study is summarized in Table S1.

### Ultrasound settings

To test the equivalent ability of BBB opening among three MBs, a single-element 0.4-MHz FUS transducer (Imasonics, Besancon, France; diameter = 60 mm, radius of curvature = 80 mm) was used to sonicate the rat brain under three power levels (MI = 0.62, 0.85, and 1.38) at a uniform MB concentration. To test the persistent ability of *in-vivo* BBB-opening among these three MBs, the other single-element 0.5-MHz FUS transducer was used with a similar exposure level to 0.62 MI. A function generator (33120 A, Agilent, Palo Alto, CA) was used to generate the driving signal to a radiofrequency power amplifier (150A100B, Amplifier Research, Souderton, PA, USA) operating in burst mode (duty cycle: 1%; PRF: 1 Hz; duration: 120 s). Animals were anesthetized with isoflurane (1–2%) and then shaved to remove the hair on their scalps. Ultrasonic gel was used to couple the ultrasound energy between the transducer and the skull. The FUS exposures were conducted in the presence of MBs at a dosage of 4 × 10^7^ bubbles/kg, with the mixed saline diluting to a total IV injection volume of 0.3 mL. The animal experimental arrangement was illustrated in Fig. S1.

In exposure-level dependent study, a single exposure was conducted per animal with the target located at 1 mm posterior to the bregma, 3 mm left to the midline and at a 5-mm depth from the dorsal brain surface. Three MBs (SonoVue, Definity, and USphere) used under three exposure levels (MI equal to 0.62, 0.85, and 1.38) to produce 9 subgroups (n = 3 per subgroup). In multiple-exposure persistence study, four exposures (MI = 0.62) were conducted with the targets located at: (1) 0.5 mm prior to the bregma, 2.5 mm right to the midline; (2) 4.5 mm posterior to the bregma, 2.5 mm right to the midline; (3) 4.5 mm posterior to the bregma, 2.5 mm left to the midline; (4) 0.5 mm prior to the bregma, 2.5 mm left to the midline. Two approaches were attempted in the multiple-exposure MBs persistence test: (1) Exposure time was fixed for the four exposures, and (2) exposure time was adaptively adjusted to compensate MBs decay. In the exposure-time fixed test (n = 5 per subgroup, for 3 subgroups; exposure level fixed to 0.62 MI), the exposures started at 0, 3, 6, 9 min after a bolus of MBs injection to sequentially sonicate the four designated brain targets with each exposure lasting two minutes. In the exposure-time adjusted test (n = 5 per subgroup, for 3 subgroups; exposure level fixed to 0.62 MI), the exposure time at the four targeted sites were adjusted to 15, 30, 60, 120 s without time-lapse.

### MRI

A 7-Tesla magnetic resonance scanner (ClinScan, Bruker, Germany) was used to acquire images to *in-vivo* monitor the FUS-induced BBB opening. Animals were secured in the center of the magnet by an acrylic holder and anesthetized with isoflurane (1–2%) during the entire MRI procedures. DCE T1-weighted imaging was performed to evaluate the permeability of the BBB. The imaging parameters were as follows: gradient- recall-echo sequence, TR/TE = 2.31 ms/0.76 ms, slice thickness = 0.8 mm, slice number = 14; matrix = 132 × 192, flip angle = 5°/10°/15°/20°/25°/30°). A total of 128 image data sets were acquired for 5 min. Upon completion of the 10th acquisition, an intravenous bolus of gadolinium (Gd-DTPA, Magnevist, Berlex Laboratories, Wayne, NJ, USA; molecular weight: 938 Da) was administered as an MRI contrast agent for dynamic acquisition. The kinetic parameter K_trans_ was estimated from post-processed DCE T1 images as described in the previous studies, with this index can effectively describe the scale of blood-to brain permeability and has been shown to serve as a valid *in-vivo* imaging index to describe BBB-opened scale^21^,^28^. A circular ROI was assigned at the targeted BBB-opening region in K_trans_ maps for statistical analysis. In addition, Susceptibility-weighted imaging (SWI) sequences were acquired to identify possible tissue hemorrhage associated with FUS-induced BBB opening using the following parameters: TR/TE = 30 ms/18 ms; flip angle = 40°; slice thickness = 0.6 mm; matrix size = 256 × 384; and FOV = 80 × 130 mm^2^.

### Evans blue quantification

Evans blue dye (3% in saline) was intravenous injected (30 mg/kg) and the animals were sacrificed two hours later (EB when administering into circulation immediately bound to serum albumin and forms a 67-kDa molecular complex which can only penetrate into brain parenchyma only when the BBB is compromised). All animals were first deeply anesthetized with 35% chloral hydrate and perfused with saline. The brains were separated along the transverse suture and brain tissues were weighed and placed in formamide (100 mg/1 mL) at 60 °C for 24 h. The sample was centrifuged for 20 min at 14,000 rpm. The concentration of dye extracted from each brain was determined spectrophotometrically at 620 nm and was compared with a standard graph created by recording optical densities from serial dilutions of EB in formamide solutions with blank brain tissues, which were cleared by centrifugation. Detailed EB quantitation method can be found in our previous studies^28^,^29^.

### Histology

EB dye was injected intravenously prior to sonication to identify the BBB-opened area for further quantification. The rats were sacrificed about 4 h after sonication and the brain samples were sequentially sectioned into 2-μm thicknesses. SWI images were acquired for the purpose of comparison. Representative sections were stained with hematoxylin and eosin (H&E). Histologic evaluation was performed blind to the ultrasound parameters; however, the observer was informed of the specific sonication side.

### Ultrasound contrast imaging

To observe MB pharmacodynamic change with a bolus administration, the ultrasound imaging was performed on a Vevo 2100 pre-clinical ultrasound scanner (VisualSonics, Toronto, ON, Canada) with a 40-MHz transducer and operated in MBs contrast-mode. To ensure sufficient energy penetration of diagnostic ultrasound, this animal group was subjected to craniotomy (while other FUS experiment groups all kept the skull intact) under the assistance of stereotactic head fixing prior to brain image scanning (n = 3). Different MBs were sequentially administered intravenously in the same animal (with the dosages all fixed to 10^8^ bubbles/kg), with at least 40 min injection interval to prevent any residual interference between MBs. Before MB injection, an image baseline was taken and then contrast data was acquired for 7 min with a total imaging scanning time of 11 min.

### Passive cavitation detection

To clarify the association between the BBB opening and MI, passive cavitation detection (PCD) was performed in an *in vitro* setup to quantify the degrees of stable cavitation and inertial cavitation caused by MBs at various acoustic pressures. The 0.4-MHz transducer was driven by an amplifier and a waveform generator (model AWG 2040, Tektronix, CA, USA) to transmit burst-FUS to an agar-based vessel phantom (2% agarose) with a diameter of 1 mm. The duty was 100 cycles and the acoustic pressures were compatible with the FUS acoustic pressures attenuated by the rat skull (0.26–0.88 MPa). For PCD, a customized focused hydrophone with a bandwidth of 0.01–10 MHz (model Y-134, Sonic Concepts Inc., WA, USA) was placed at a 90 degree angle to receive characteristic signals induced by the stable cavitation (SC) and inertial cavitation (IC) of different bubbles. The received signals were digitized using an oscilloscope (model LT354, LeCroyCorp., NY, USA) after being amplified by a broadband receiver (BR-640A, Ritec, Warwick, RI, USA). The signals were converted to the frequency domain using MATLAB software (Mathworks, Natick, MA, USA) for assessment of SC and IC. For each case, the SC dose (SCD) was computed with the integration of the subharmonic frequency band at 0.2 MHz for 0.4-MHz FUS. The IC dose (ICD) was computed with the integration of multiple frequency bands that fell within the bandwidth of the focused hydrophone but outside the incident, subharmonic, harmonic, and ultraharmonic frequencies of FUS. For both SCD and ICD, the calculated results were normalized to the results of pure water obtained under the same experimental conditions. The experimental setup and quantification method are detailed described previously^30^,^31^.

### Statistical analysis

All the data were presented as mean ± standard deviation (SD). Statistical analysis was performed on a computer using SPSS 20.0 software (SPSS Inc., Chicago, IL, USA) by two-way ANOVA with the post-hoc Dunnet test or Mann-Whitney U test where appropriate. Comparison was considered to be statistically significant when *p* < 0.05 (denoted as “*”).

## Additional Information

**How to cite this article**: Wu, S.-K. *et al*. Characterization of Different Microbubbles in Assisting Focused Ultrasound-Induced Blood-Brain Barrier Opening. *Sci. Rep.* **7**, 46689; doi: 10.1038/srep46689 (2017).

**Publisher's note:** Springer Nature remains neutral with regard to jurisdictional claims in published maps and institutional affiliations.

## Supplementary Material

Supplementary Information

## Figures and Tables

**Table 1:** Characteristics of three different microbubbles used in this study.

Category	Gas	Shell	Mean Size (μm)	Concentration (bubbles/mL)	Injected dosage (bubbles/kg)
SonoVue^TM^	SF_6_	Lipid	2.5	2 × 10^8^	4 × 10^7^
Definity^TM^	C_3_F_8_	Lipid	1.5	1.0 × 10^10^	4 × 10^7^
USphere^TM^	C_3_F_8_	Lipid	0.85	2.8 × 10^10^	4 × 10^7^
