# Supplementary material for: Characterization of Different Microbubbles in Assisting Focused Ultrasound-Induced Blood-Brain Barrier Opening
Source: Sci Rep. 2017 Apr 20;7:46689. doi: 10.1038/srep46689 (PMC5397978; doi:10.1038/srep46689)
Supplement: Supplementary Information [file srep46689-s1.doc]

**Characterization of Different Microbubbles in Assisting Focused Ultrasound-Induced Blood-Brain Barrier Opening**

Sheng-Kai Wu^1§^, Po-Chun Chu^2,3§^, Wen-Yen Chai^3,4^, Shih-Tsung Kang^5^, Chih-Hung Tsai^3^, Ching-Hsiang Fan^5^, Chih-Kuang Yeh^5*^, Hao-Li Liu^3,6,7*^

^1^Institute of Biomedical Engineering, College of Medicine and College of Engineering, National Taiwan University, Taipei, Taiwan

^2^Department of Research and Development, NaviFUS corp., Taipei, Taiwan

^3^Department of Electrical Engineering, Chang-Gung University, Taoyuan, Taiwan

^4^Department of Diagnostic Radiology and Intervention, Chang-Gung Memorial Hospital, Taoyuan, Taiwan,

^5^Department of Biomedical Engineering and Environmental Sciences, National Tsing Hua University, Hsinchu, Taiwan

^6^Department of Neurosurgery, Chang Gung Memorial Hospital, Taoyuan, Taiwan

^7^Medical Imaging Research Center, Institute for Radiological Research, Chang Gung University and Chang Gung Memorial Hospital, Taoyuan, Taiwan.

^§^S.-K. Wu and P.-C. Chu contributed equally to the work.

^*^ Corresponding authors: E-mail: ckyeh@mx.nthu.edu.tw (C.-K. Yeh), haoliliu@mail.cgu.edu.tw (H.-L. Liu).

Supplementary Information

**Table S1.** Summary of in-vivo animal experiments. A total of 66 male Sprague Dawley rats were employed in this study. The contrast-enhanced ultrasonography test was conducted with n=3 per group (totally n = 9). In the MI-dependence test, three MBs (SonoVue, Definity, and USphere) were employed under three exposure levels (MI of 0.62, 0.85, and 1.38) with n = 3 per group (totally n = 27). In the persistence test, the experiments include fixed and adjusted exposure times with n = 5 per group (totally n = 30).

|  | Contrast-enhanced ultrasono-graphy | MI-dependence test,  Single-exposure | | | Persistence test, multiple exposure (MI=0.62) | |
| --- | --- | --- | --- | --- | --- | --- |
| Exposure condition (MI/ sec) | -- | 0.62/ 120 | 0.85/ 120 | 1.38/ 120 | 0.62/  120, 120, 120, 120 | 0.62/  15, 30,  60, 120 |
| SonoVue | 3 | 3 | 3 | 3 | 5 | 5 |
| Definity | 3 | 3 | 3 | 3 | 5 | 5 |
| USphere | 3 | 3 | 3 | 3 | 5 | 5 |

**
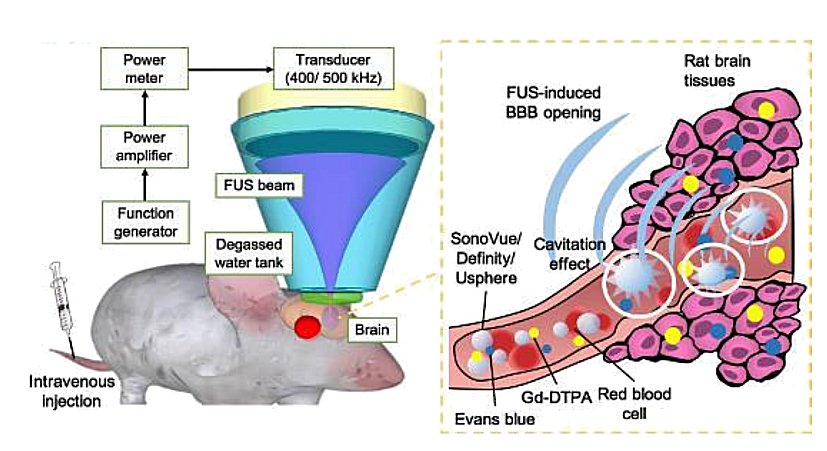
**

**Fig. S1.** Schematic representation of blood-brain barrier (BBB) opening by focused ultrasound (FUS) sonication after intravenous injection of microbubbles (SonoVue, Definity, or Usphere). Evans blue dye and Gd-DTPA are used to evaluate the degree of BBB opening.


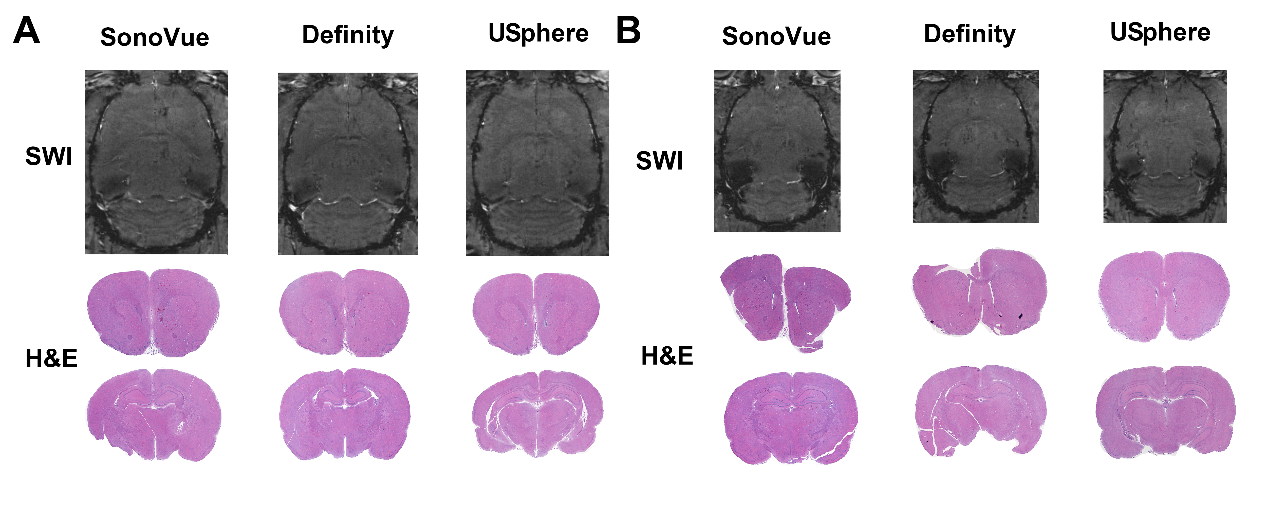


**Fig. S2.**  MR-SWI images and their corresponding HE-stained brain sections for four 120s sonication spots and time-adjusted sequences.


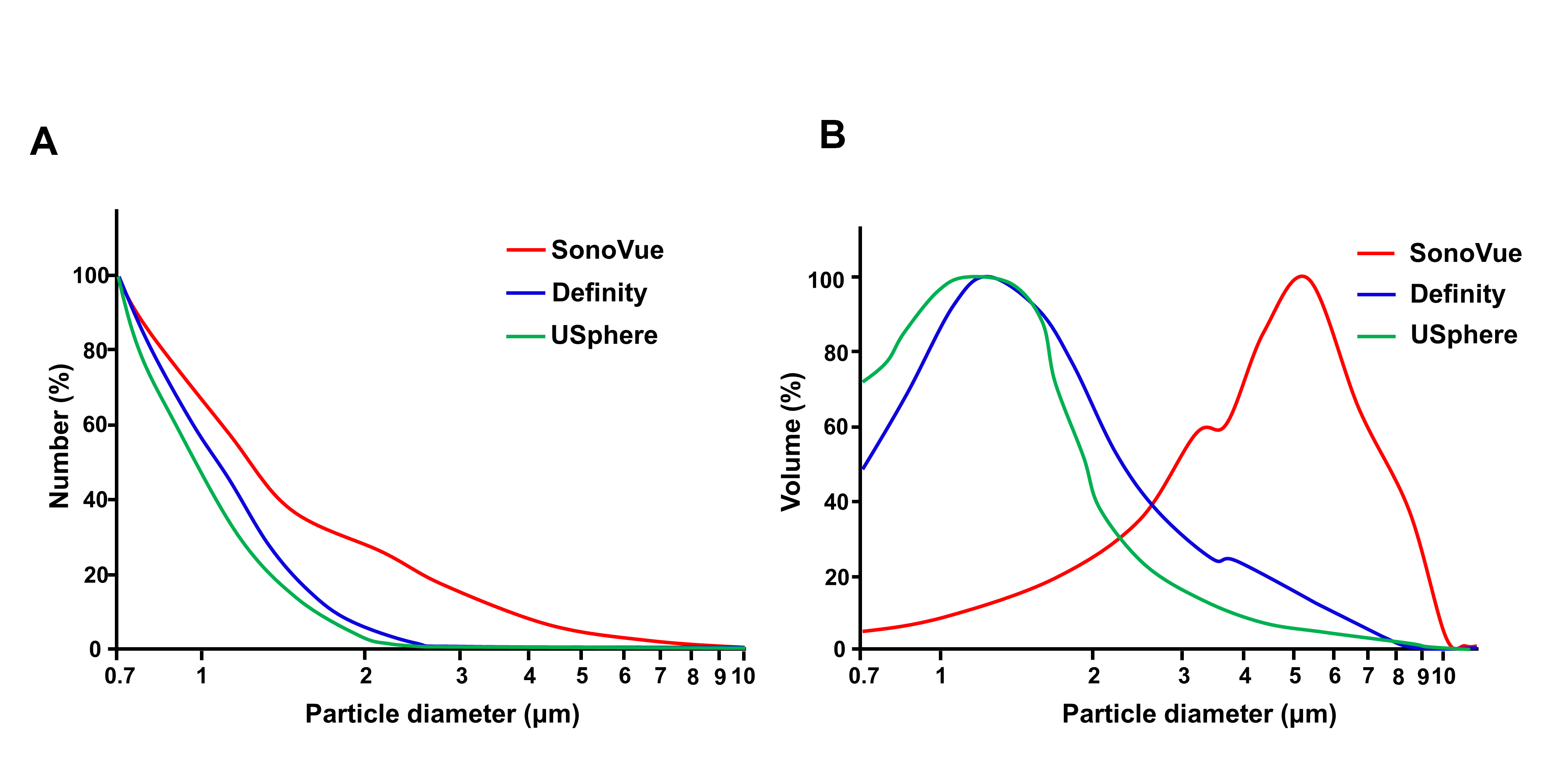


**Fig. S3.** Size distribution of three different microbubble types used in this study. (A) number-weighted size distributions (in %); (B) volume-weighted size distributions (in %).
